# Metatranscriptomes Reveal That All Three Domains of Life Are Active but Are Dominated by Bacteria in the Fennoscandian Crystalline Granitic Continental Deep Biosphere

**DOI:** 10.1128/mBio.01792-18

**Published:** 2018-11-20

**Authors:** Margarita Lopez-Fernandez, Domenico Simone, Xiaofen Wu, Lucile Soler, Emelie Nilsson, Karin Holmfeldt, Henrik Lantz, Stefan Bertilsson, Mark Dopson

**Affiliations:** a Centre for Ecology and Evolution in Microbial Model Systems (EEMiS), Linnaeus University, Kalmar, Sweden; b Department of Medical Biochemistry and Microbiology, Uppsala University, NBIS, Science for Life Laboratory, Uppsala, Sweden; c Department of Ecology and Genetics, Limnology and Science for Life Laboratory, Uppsala University, Uppsala, Sweden; CEH-Oxford

**Keywords:** metatranscriptomes, mRNA, rRNA, deep biosphere, groundwaters, metatranscriptomes

## Abstract

The continental subsurface is suggested to contain a significant part of the earth’s total biomass. However, due to the difficulty of sampling, the deep subsurface is still one of the least understood ecosystems. Therefore, microorganisms inhabiting this environment might profoundly influence the global nutrient and energy cycles. In this study, *in situ* fixed RNA transcripts from two deep continental groundwaters from the Äspö Hard Rock Laboratory (a Baltic Sea-influenced water with a residence time of <20 years, defined as “modern marine,” and an “old saline” groundwater with a residence time of thousands of years) were subjected to metatranscriptome sequencing. Although small subunit (SSU) rRNA gene and mRNA transcripts aligned to all three domains of life, supporting activity within these community subsets, the data also suggested that the groundwaters were dominated by bacteria. Many of the SSU rRNA transcripts grouped within newly described candidate phyla or could not be mapped to known branches on the tree of life, suggesting that a large portion of the active biota in the deep biosphere remains unexplored. Despite the extremely oligotrophic conditions, mRNA transcripts revealed a diverse range of metabolic strategies that were carried out by multiple taxa in the modern marine water that is fed by organic carbon from the surface. In contrast, the carbon dioxide- and hydrogen-fed old saline water with a residence time of thousands of years predominantly showed the potential to carry out translation. This suggested these cells were active, but waiting until an energy source episodically becomes available.

## Abstract (precis)

A newly designed sampling apparatus was used to fix RNA under *in situ* conditions in the deep continental biosphere and benchmarks a strategy for deep biosphere metatranscriptomic sequencing. This apparatus enabled the identification of active community members and the processes they carry out in this extremely oligotrophic environment. This work presents for the first time evidence of eukaryotic, archaeal, and bacterial activity in two deep subsurface crystalline rock groundwaters from the Äspö Hard Rock Laboratory with different depths and geochemical characteristics. The findings highlight differences between organic carbon-fed shallow communities and carbon dioxide- and hydrogen-fed old saline waters. In addition, the data reveal a large portion of uncharacterized microorganisms, as well as the important role of candidate phyla in the deep biosphere, but also the disparity in microbial diversity when using standard microbial 16S rRNA gene amplification versus the large unknown portion of the community identified with unbiased metatranscriptomes.

## INTRODUCTION

Microbial life in the deep subsurface has been intensely studied over the last two decades, but the deep subsurface is still one of the least understood environments on earth. Investigations carried out in upper oceanic crust fluids (1–3), deeply buried marine sediments (4–6), terrestrial sedimentary rocks (7–9), and granitic groundwaters (10, 11) indicate that microorganisms are widespread. It is estimated that the deep continental subsurface contains from 10^16^ to 10^17^ g biomass carbon (12, 13), and a critical question for these highly oligotrophic environments is which of these microorganisms are active or dormant (14).

In continental groundwaters, microbial abundance and activity are strongly positively correlated to the proximity of the photosynthesis-fueled surface (15), and thus, water-bearing deep fracture systems are extremely oligotrophic (16). Nevertheless, several studies support that at least a portion of the deep biosphere community is viable. This evidence includes active sulfur-driven denitrifiers in quartzite and shale (17) and a hydrogen-driven microbial community in Opalinus clay rock (18). Moreover, the detection of ATP (19), the presence of phages (20, 21), changes in the community structure (22), and the capacity for biofilm formation (11) in granitic hard rock support that continental crystalline rock communities are active. A recent study confirms the presence of viable taxa in a deep continental crystalline rock (23), where almost half of the cells are smaller than 0.2 µm and have relatively small genomes (24), and many of them are from candidate phyla (25, 26). This suggests that small cell size and small genomes might be adaptations to the oligotrophic conditions (24), where any nonviable cells are rapidly degraded and recycled into new biomass (23). However, it is unknown which populations within the crystalline rock subsurface communities are active under *in situ* conditions and the specific metabolic processes that they carry out.

High-throughput sequencing allows for reconstruction of entire microbial communities, and its application for metatranscriptomics provides the means for tracking the metabolic activity of microbial communities as they occur in nature (27). Studies of microbial metabolic activity in marine deep sea sediments via metatranscriptomics have revealed that the collective activity of the subsurface microbiota differs according to sediment depth, organic matter age, and geochemical zones (28, 29).

Despite the suggested importance of the deep biosphere for global biogeochemical cycles and anthropogenic activities, difficulties in obtaining valid samples have resulted in poor understanding of the metabolic activities of biota in the crystalline rock deep biosphere. This study was carried out at the Swedish Nuclear Fuel and Waste Management Company-operated Äspö Hard Rock Laboratory (Äspö HRL), an underground geosphere laboratory extending to a depth of 460 m below sea level (mbsl) (30) (see Fig. S1A in the supplemental material). The Äspö HRL is located in Proterozoic crystalline bedrock (31), and its geology, chemistry, and hydrology have been described previously (32–34). Accessing the deep continental biosphere from such an underground laboratory constructed over 20 years ago helps to circumvent the contamination often associated with drilling or excavation, and it also provides access to boreholes that interconnect with fracture systems bearing waters that flow into the tunnel by pressure rather than pumping (35).

10.1128/mBio.01792-18.2 FIG S1 (A) Äspö HRL tunnel map showing the two different borehole water types sampled. (B) Design (left) and the finished sampling vessel (right) for preservation of RNA from borehole water maintained under *in situ* conditions. Download FIG S1, TIF file, 2.6 MB. Copyright © 2018 Lopez-Fernandez et al. 2018 Lopez-Fernandez et al. This content is distributed under the terms of the Creative Commons Attribution 4.0 International license.

To circumvent issues of changes in cellular RNA transcript levels with extended cell capture times, a novel sampling device to rapidly fix cells for collection of RNA transcripts under *in situ* conditions was designed. Subsequently, RNAs from two Äspö HRL groundwaters of contrasting ages and characteristics were converted to cDNA and subjected to high-throughput sequencing. This study is the first to identify the active microbial community composition and the processes they carry out in contrasting continental groundwaters of different ages and chemical characteristics.

## RESULTS

### Active community members from all three domains of life.

**FIG 1:**
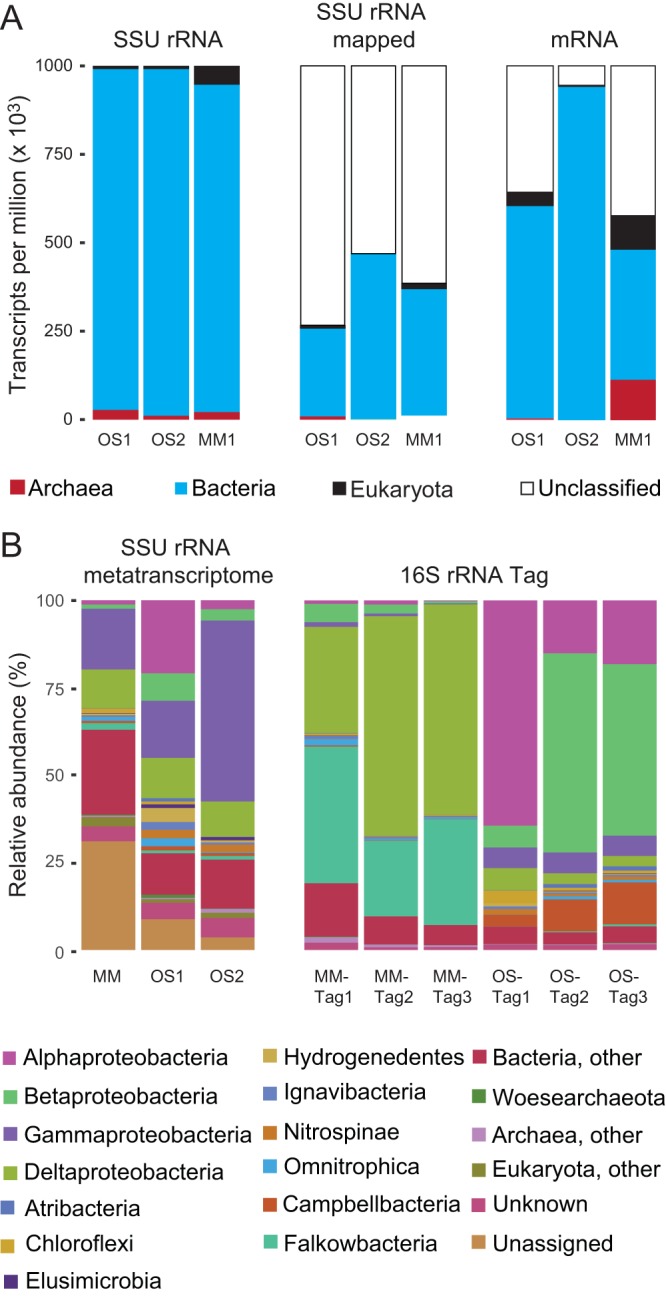
Active deep biosphere communities inferred from metatranscriptome data. (A) Distribution of SSU rRNA reads based on cmsearch of domain-level covariance models available on Rfam. SSU rRNA reads were mapped to the reconstructed SSU rRNA contigs (≥300 bp and ≥5 average coverage) whose phylogenetic placement was assessed by the RAxML evolutionary placement algorithm (EPA), while the mRNA transcripts were given a taxonomic assignment using Kaiju. (B) Total prokarytic and eukaryotic community based on SSU rRNA gene amplicon sequencing and the active portion according to SSU rRNA gene transcripts from the metatranscriptome. Phylogenetic assignment was carried out at the phylum level, including the most recent candidate phyla (26, 37), except that the Proteobacteria were split into classes. Only phyla identified in the three samples with >0.01% relative abundance were included, and the remaining rare lineages were included in “other.” “Unknown” refers to tree nodes with poor taxonomic information and “Unassigned” to SSU transcripts that could not be reliably placed on the reference tree.

**FIG 2:**
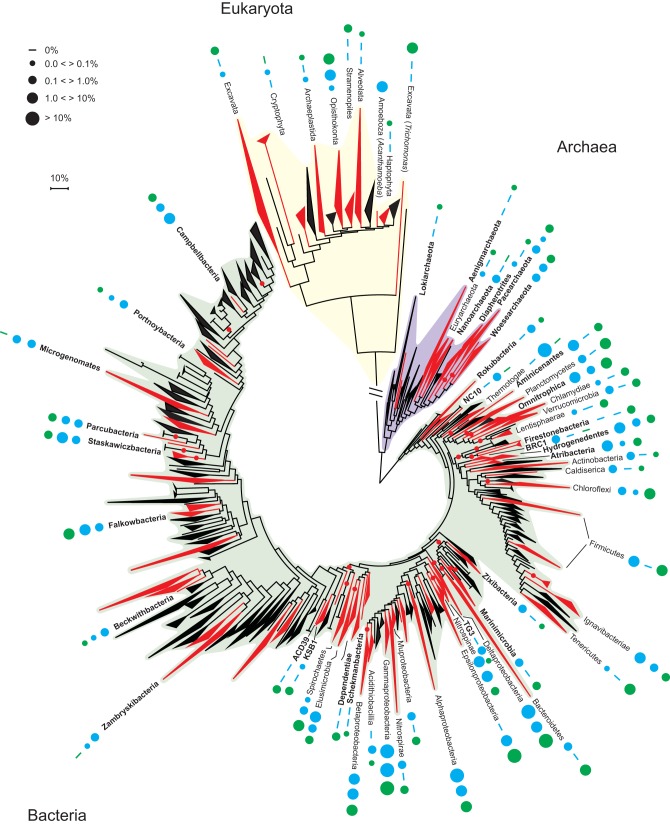
Diversity of active community members from all three domains. The reconstructed SSU rRNA gene contigs were placed on the tree of life using RAxML-EPA. The tree includes the most recent candidate phyla (26, 37) and was inferred by RAxML using the GTRCAT evolutionary model. Where possible, the leaves were collapsed into phyla, except for the Proteobacteria, which are shown in classes. The colored circles denote the origin of the sequences from OS (blue; OS1 in the inner blue ring and OS2 in the outer blue ring) and MM (green; MM1) waters. The circle size relates to the TPM of domain-level SSU rRNA distribution. Only bacterial phyla with a TPM distribution higher than 0.1% in at least one of the waters are named in the tree. Candidate phyla are labeled in bold, and SSU rRNA transcripts that could not be placed in specific phyla are shown by red circles. The scale bar shows 10% sequence divergence.

Phylogenetic placement of SSU rRNA transcripts *de novo* assembled from data sets showed that lineages encompassing all three domains of life (Fig. 1 and 2) were detected in the “modern marine” (MM) and “old saline” (OS) waters. However, Bacteria showed a clear dominance over Archaea and Eukarya. In addition, transcripts accounting for 3% to 31% of total small subunit (SSU) rRNA abundance (shown as “Unassigned” in Fig. 1B) could not be confidently placed onto the reference tree, which included recently described candidate phyla (Fig. 1 and 2). Comparative analysis of Bacteria and Archaea domain rRNA transcripts and 16S rRNA gene amplicons showed that almost all SSU transcripts had a matching amplicon, but not vice versa (Fig. 1; see Data Set S1 in the supplemental material).

10.1128/mBio.01792-18.9 DATA SET S1 Phylogenetic placements of reconstructed SSU rRNA gene contigs and tag sequences used to perform the RAxML-EPA phylogenetic placement reported in Fig. 2 in Newick format. Download Data Set S1, TXT file, 0.3 MB. Copyright © 2018 Lopez-Fernandez et al. 2018 Lopez-Fernandez et al. This content is distributed under the terms of the Creative Commons Attribution 4.0 International license.

**FIG 3:**
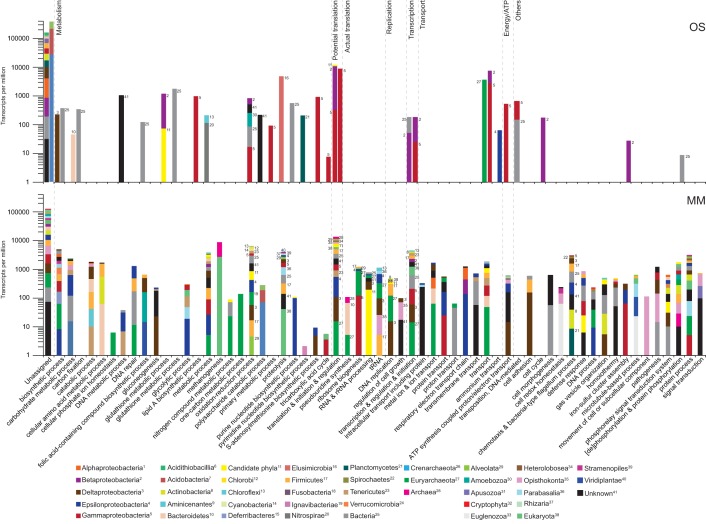
Gene expression profiles in the deep biosphere. Transcripts annotated to Gene Ontology (GO) “Biological Process” terms are shown for OS (OS1, left-hand bar; OS2, right-hand bar) and MM (MM1) waters. All GO processes for which mRNA transcripts were identified in at least one sample were included, and transcripts not assigned to any GO processes were named “Unassigned.” GO processes with TPM assigned to a phylum (or other taxonomical clasiffication) of less than 1% of the total for that term were amalgamated as Bacteria, Archaea, or Eukarya. Superscripts identify the respective classification included in the main text and metabolic model.

In line with the SSU rRNA data, the mRNA transcripts supported activity of all three domains of life in both waters, again with a clear dominance of Bacteria (Fig. 1; see Table S1 in the supplemental material). The majority of the mRNA transcripts were not functionally classified in either of the two aquifers (Fig. 1 and 3). mRNA transcripts were mapped back to previously generated metagenome assembled genomes (MAGs) from the same water types analyzed in this study (24), but only a few matches mapped to viruses (data not shown). In addition, only 6.0, 0.0, and 29.1% of the mRNA transcripts assigned to candidate phyla in the OS1, OS2, and MM1 samples, respectively, could be annotated to Gene Ontology (GO) “Biological Process” terms. The total mRNA transcripts were assigned to 29 and 220 different processes in the OS and MM waters, respectively (see Table S2 in the supplemental material). Finally, mapping the SSU rRNA and mRNA reads to the previously generated MAGs in Wu et al. (24) suggested that small (passing through a 0.22-µm-pore membrane) and large cells were active in the two deep aquifer communities (see Table S3 in the supplemental material).

10.1128/mBio.01792-18.3 TABLE S1 SSU rRNA gene and mRNA transcript abundances for domain, phyla, and other classification levels. Download Table S1, XLSX file, 0.1 MB. Copyright © 2018 Lopez-Fernandez et al. 2018 Lopez-Fernandez et al. This content is distributed under the terms of the Creative Commons Attribution 4.0 International license.

10.1128/mBio.01792-18.4 TABLE S2 mRNA transcript abundances related to “Processes” as annotated using GO “Biological Process” terms. Download Table S2, XLSX file, 0.1 MB. Copyright © 2018 Lopez-Fernandez et al. 2018 Lopez-Fernandez et al. This content is distributed under the terms of the Creative Commons Attribution 4.0 International license.

10.1128/mBio.01792-18.5 TABLE S3 16S rRNA and mRNA transcript reads mapped back to metagenome assembled genomes (MAGs) from the same water types analyzed in this study. Download Table S3, XLSX file, 0.1 MB. Copyright © 2018 Lopez-Fernandez et al. 2018 Lopez-Fernandez et al. This content is distributed under the terms of the Creative Commons Attribution 4.0 International license.

### Active candidate lineages.

Many uncultured candidate phyla actively expressed genes in the extremely oligotrophic, granitic continental deep subsurface investigated here (Fig. 3). Candidate phyla represented 18.3, 6.8, and 10.8% of the total abundance of the Archaea and Bacteria domains (Table S1) in the duplicate OS and single MM metatranscriptomes, respectively.

The OS and MM waters contained many bacterial lineages defined within candidate phyla, totaling 4, 4.5, and 3.7% of the total abundance in the OS1, OS2, and MM1 samples, respectively (Fig. 2; Data Set S1 and Table S1). Of these, Campbellbacteria, Portnoybacteria, and Beckwithbacteria were predominantly active in the OS samples, with Falkowbacteria active in both OS and MM waters. In addition, metabolically resolved candidate phyla grouping in other parts of the phylogenetic tree included Schekmanbacteria (only identified in the MM water) and Marinimicrobia (identified in the OS1 and MM1 samples).

The dominant active Archaea in both water types encompassed members affiliated with the candidate phyla Woesearchaeota and Pacearchaeota, totaling 11,218, 1,368, and 5,394 transcripts per million (TPM) in the OS1, OS2, and MM1 samples, respectively (Fig. 2). In addition, OS1 and MM1 samples contained SSU rRNA transcripts that were placed within Euryarchaeota (155 and 459 TPM, respectively) and Diapherotrites (176 and 268 TPM). The MM water also contained SSU rRNA gene transcripts that mapped with Aenigmarchaeota (246 TPM) and Lokiarchaeota (191 TPM). Finally, only the OS1 sample contains SSU rRNA gene transcripts that mapped with Nanoarchaeota (1,384 TPM).

### Active cultured lineages.

Although lineages containing cultured representatives accounted for less than half of the phyla detected in the two water types, they constituted the majority of the SSU rRNA reads that could be mapped (66, 81, and 53% for the OS1, OS2, and MM1 samples, respectively). These bacteria were dominated by the *Alpha*-, *Delta*-, *Gamma*-, and Betaproteobacteria in the OS water compared to *Gamma*-, *Delta*-, and Epsilonproteobacteria in the MM water (Fig. 2). In the OS water, the dominant Betaproteobacteria aligned with Thiobacillus denitrificans (49,626 and 18,325 TPM), along with many transcripts that aligned with Deltaproteobacteria (26,593 and 41,021 TPM), concretely with families Desulfobulbaceae, Desulfobacteraceae, and Syntrophaceae. The MM water also contained many SSU rRNA transcripts assigned to Deltaproteobacteria (32,559 TPM) and Pseudomonadaceae (56,232 TPM). In addition, the MM water contained transcripts from an Epsilonproteobacteria most closely affiliated with Sulfurimonas denitrificans (51,759 TPM) and Arcobacter nitrofigilis (49,142 TPM).

### Active *Eukarya*.

Here we reveal the total active Eukarya community members from two different deep groundwater crystalline bedrock fracture waters (Fig. 2). These Eukarya partially differed in the two water types, with the dominant SSU rRNA gene sequences present in the OS water aligning with the Acanthamoeba genus (9,293 and 0 TPM for each of the two OS replicates). In addition, other SSU rRNA gene sequences were from members of the Opisthokonta (386 and 14,050 TPM), Cryptophyta (0 and 466 TPM), and Excavata (132 and 0 TPM). Finally, one sequence aligned close to the root of the Archaeplastida (180 and 0 TPM). The MM water contained SSU rRNA gene sequences placed within the Opisthokonta (12,063 TPM), Stramenopiles (1,986 TPM), and Acanthamoeba genus (412 TPM). The results report for the first time in the deep biosphere SSU rRNA gene transcripts from the Excavata supergroup (11,701 TPM), of which more than 57% were from the genus Trichomonas. In addition, sequences from Archaeplastida (114 TPM), Alveolata (61 TPM), and Haptophyta (27 TPM) were identified.

### Metabolic processes in the Äspö HRL crystalline rock deep continental biosphere.

**FIG 4:**
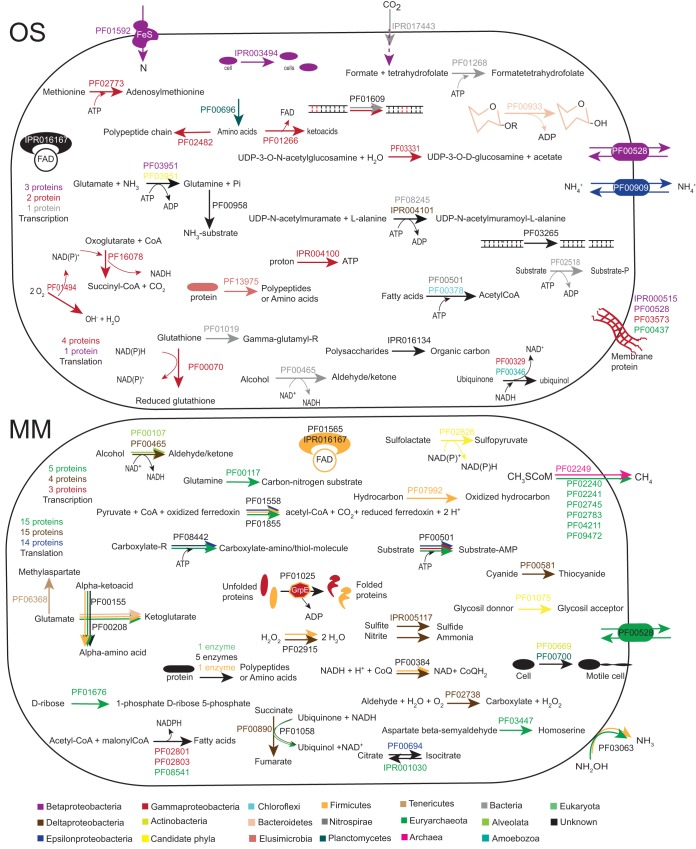
Metabolic model representing the active processes in the OS and the MM waters. Processes were based upon Gene Ontology (GO) terms and include the relevant Pfam and InterProScan identifications. Processes with TPM assigned to a phylum (or other taxonomical classification) are color coded as described in the legend to Fig. 3. Only major processes are shown for the MM water, while all identified processes have been included for the OS water.

The mRNA transcripts annotated with GO “Biological Process” terms assigned to characterized Bacteria lineages in the OS water (Fig. 3 and 4; Table S2) included the *Beta*- (26,027 and 312,625 TPM), *Alpha*- (21,157 and 7 TPM), *Gamma*- (15,024 and 80,867 TPM), and Deltaproteobacteria (13,282 and 147 TPM), along with Planctomycetes (7,986 and 19 TPM) and Actinobacteria (7,383 and 1,989 TPM); candidate phyla were also represented by the Desantisbacteria (1,080 and 0 TPM) and Atribacteria (21 and 0 TPM), Archaea by the phylum Euryarchaeota (3,561 and 0 TPM), and Eukarya lineages from Opisthokonta (247 and 801 TPM), Amoebozoa (170 and 0 TPM), Alveolata (0 and 33 TPM), and Rhodophyta (13 and 0 TPM). Based on mRNA gene ontologies, the OS community was dominated by transcripts encoding translational potential (translational initiation, elongation, and termination), with 11,097 and 8,881 TPM assigned to the Proteobacteria in the OS1 and OS2 samples, respectively (Fig. 3 and 4; Table S2). However, no mRNA transcripts were identified for actual translation, DNA replication, or regulation of cell growth in either of the OS1 and OS2 samples. mRNA transcripts for ribulose bisphosphate carboxylase (RuBisCO) in the OS1 replicate were assigned to the bacterial domain (345 TPM). mRNA transcripts encoding transport processes, glutamine synthetase in nitrogen metabolism, and oxidation-reduction processes were also assigned to Betaproteobacteria. mRNA transcripts assigned to Gammaproteobacteria and potentially the active Pseudomonadaceae included two subunits of the respiratory chain NADH dehydrogenase. In contrast, a high proportion of mRNA transcripts were for proteolysis (4,784 and 0 TPM), including Gag polyprotein putative aspartyl protease, which was assigned to the Elusimicrobia. Finally, the OS1 replicate had mRNA transcripts assigned to DNA-mediated transposition (666 TPM).

The MM1 sample had a much more diverse active community, with mRNA transcripts assigned to a large variety of GO processes (Fig. 3 and 4; Table S2). The majority of the mRNA transcripts were assigned to the Bacteria domain, including 27 candidate phyla, such as Desantisbacteria (1,610 TPM), Atribacteria (625 TPM), Riflebacteria (384 TPM), Schekmanbacteria (321 TPM), Omnitrophica (225 TPM), Ryanbacteria (201 TPM), and Parcubateria (181 TPM). In addition, mRNA transcripts were identified from the characterized bacterial classes *Delta*- (15,603 TPM), *Gamma*- (13,117 TPM), and Epsilonproteobacteria (12,243 TPM), Bacteria domain (12,167 TPM), and Firmicutes phylum (9,739 TPM). mRNA transcripts were assigned to 7 Archaea phylogenetic classifications, primarily from the Euryarchaeota phylum (32,130 TPM) and Archaea domain (1,542 TPM). Finally, mRNA transcripts were assigned to 13 phylogenetic classifications within the Eukarya domain, mainly from the Opisthokonta group (8,060 TPM), Eukarya domain (4,584 TPM), Parabasalia class (2,738 TPM), Alveolata superphylum (2,652 TPM), and Stramenopiles superphylum (2,247 TPM). The general higher activity of the cells in the MM water was reflected by mRNA transcripts for transcription assigned to Euryarchaeota and *Delta*- and Gammaproteobacteria, along with translation assigned to Methanobrevibacter-like and Desulfotalea-like populations. Further mRNA transcripts included a zinc-binding dehydrogenase assigned to a Perkinsus-like population, molybdopterin oxidoreductases assigned to Firmicutes and Epsilonproteobacteria, a hydroxyacid dehydrogenase assigned to the candidate phylum Atribacteria, and flagellum-dependent cell motility assigned to Planctomycetes and Actinobacteria.

## DISCUSSION

In this study, SSU rRNA data and mRNA transcripts supported activity of all three domains of life, with a clear dominance of bacteria in both investigated waters from Äspö HRL (Fig. 1 and 2). Even though using SSU rRNA transcripts as a proxy for growth and/or activity has been questioned (36), here we use the term “active” as meaning a “protein synthesis potential.” It is possible that the amplified SSU amplicon sequences were from dead cells, although a study using propidium monoazide that binds to DNA and inhibits subsequent amplification revealed that most of the cells have an intact cell membrane. This confirmed that lysed cells are rapidly recycled into living biomass in this extremely oligotrophic environment (23). In addition, that almost all SSU transcripts had a matching 16S rRNA gene amplicon but not vice versa (Fig. 1B), suggested that only a subset of the total microbial community was active at the time of sampling. Finally, that up to 31% of the total SSU rRNA transcripts were not confidently placed onto a reference tree (Fig. 1B and 2) might indicate that those unassigned SSU transcripts could represent novel candidate phyla, known phyla with no sequenced genomes, or could alternatively lack sufficient phylogenetic signal to be placed. Moreover, the greater proportion of unassigned SSU rRNA gene transcripts from the metatranscriptome compared to the 16S rRNA gene amplicons suggested that a large portion of the active prokaryotic community (Eukarya SSU rRNA gene transcripts were removed) was potentially amplified at lower efficiency by standard universal primers and thus was likely overlooked in earlier studies.

Recent advances in environmental genomics have led to the description of a large number of previously unrecognized and uncultured candidate phyla (26, 37, 38). To most confidently identify these candidate populations, RNA should be mapped to MAGs obtained from the same environment. However, as the data were compared to existing databases, this limits the possibility to identify novel active populations. Many uncultured candidate phyla actively expressed genes in these extremely oligotrophic, granitic continental deep subsurface waters (Fig. 3). The archaeal candidate phyla Woesearchaeota and Pacearchaeota were previously identified in amplicon data from these MM and OS groundwaters (24), and they have also been identified in a shallow, 6-m-deep aquifer (25). Although it is uncertain to draw inferences on metabolic capabilities from SSU rRNA similarities, both lineages lack many biosynthetic pathways and are suggested to be involved in symbiotic or fermentative lifestyles based on carbon and hydrogen metabolism (25, 39). The OS1 and MM1 samples contained SSU rRNA transcripts from Diapherotrites, which are syntrophic archaea involved in acetate production via fermentation (25). The OS1 sample also contains SSU rRNA gene transcripts that mapped with Nanoarchaeota, which have been recently isolated from a terrestrial geothermal environment (40). Many of the bacterial candidate phyla identified in both waters have streamlined genomes and lack complete tricarboxylic acid (TCA) cycles and electron transport systems, as well as missing some biosynthetic pathways (24, 26). As such, they are suggested to be fermentative and potentially live as symbionts and this may be an adaptation strategy to the oligotrophic conditions in the two waters.

The greater estimated abundance of cultured lineages that were mapped was potentially due to higher representation of such phyla in the reference phylogeny. However, it is likely that many of these cultured deep biosphere populations represented novel species that have not been isolated. The OS water had SSU rRNA transcripts aligning with Thiobacillus denitrificans, a facultative anaerobe that couples the oxidation of inorganic sulfur compounds to nitrate reduction (41). This taxon has previously been identified in MAGs from two old saline Äspö HRL waters (11, 24). The MM water also contained many SSU rRNA transcripts assigned to sulfur- and sulfate-reducing Deltaproteobacteria, Sulfurimonas denitrificans, which is a hydrogen and sulfur compound-oxidizing nitrate reducer previously identified at the Äspö HRL (22), and Arcobacter nitrofigilis, which is a nitrogen-fixing heterotroph.

Here we also reveal the total active Eukarya community members from two different deep crystalline bedrock fracture waters (Fig. 2). Eukarya have previously been identified from deep fracture water in South African deep mines (42, 43) and deep continental crystalline rocks (44). In addition, active community members have been identified from the deep marine subsurface (29, 45), and active fungi have been identified by internal transcribed spacer (ITS) sequencing of cDNA from deep granitic fracture waters (46). The dominant SSU rRNA gene sequences in the OS water aligned with the Acanthamoeba genus, which has a bacteriovorus lifestyle (47), Opisthokonta, which has been detected in deep bedrock fractures of the Fennoscandian shield (46), and Cryptophyta, which has been identified as bacterivores (48). The greater representation and diversity of eukaryotic SSU rRNA in the MM water with transcripts from Opisthokonta, Stramenopiles, Acanthamoeba, Excavata, Archaeplastida, Alveolata, and Haptophyta suggested a more important role of Eukarya in this water than in the OS community.

Previous models of microbial communities in deep crystalline rock groundwaters convey the paradigm that the communities are independent from the photosynthesis-fueled surface and are instead sustained via the “geogases” hydrogen and carbon dioxide (49). Although the MM water is suggested to be influenced by Baltic Sea water infiltration, the active microbial community was largely distinct from a shallow (0- to 1-cm-deep) anoxic sediment from a Baltic Sea coastal bay close to the Äspö HRL (50). The differences between the communities were highlighted by the relative lack of unassigned SSU transcripts and representatives from candidate phyla in the coastal shallow sediment. However, some similarities occurred with, e.g., the presence and activity of gene transcripts attributed to Sulfurimonas spp. in both the present and a previous study of the Äspö HRL (22) compared to a shallow Baltic Sea anoxic sediment (50). This suggested that even the MM and particularly the OS waters had microbial communities that had been selected by percolation from the surface and long-term isolation in the oligotrophic deep continental biosphere.

Typical of environmental samples, the majority of the mRNA transcripts were not functionally classified in either of the two MM and OS waters (Fig. 1 and 3), but this trend was likely accentuated by the many candidate phyla and potential novel adaptations to life in the deep biosphere. The observation that many GO processes in the OS water were only present in the OS1 replicate (e.g., carbon fixation, gluconeogenesis, and primary metabolic processes and oxidation reduction processes) and the low number of mRNA transcripts observed in OS2 sample support that the OS1 cells had been recently exposed to an electron donor. Due to the paucity of mRNA transcripts in the OS water, it was difficult to assign many roles to individual taxa. mRNA transcripts for RuBisCO supported that the Calvin-Benson-Bassham (CBB) cycle was the dominant carbon dioxide fixation pathway (11, 24). Although it is not essential for nitrogen fixation and other *nif* genes were not identified, transcripts for *nifU* suggested potential nitrogen fixation by Betaproteobacteria, supporting that this class was active in the OS water. mRNA transcripts for proteolysis assigned to Elusimicrobia supported that candidate phyla were recycling nutrients in response to the extremely oligotrophic conditions (23). Finally, mRNA transcripts assigned to DNA-mediated transposition suggested that cells could carry out horizontal gene transfer or genome rearrangement in this highly oligotrophic environment.

In the case of the MM water, the community was much more diverse, with mRNA transcripts assigned to a large variety of GO processes, likely due to nutrient recharge from the Baltic Sea (Fig. 3 and 4; Table S2). The absence of mRNA transcripts for carbon dioxide fixation and the few transcripts for nitrogen fixation (assigned to Euryarchaeota, Bacteria, and Atribacteria) support that the MM community was fed by carbon and bioavailable nutrients originating from the surface. However, it is plausible that autotrophic methanogenesis still occurred as mRNA transcripts for a methyl-coenzyme M reductase beta subunit (*mcrA* gene) could be assigned to a Methanobacterium-like population and a highly active Methanobrevibacter-like population. In addition, some members of the Desantisbacteria have enzymes from the Wood-Ljungdahl pathway, suggesting they may grow autotrophically (51), but transcripts coding for these genes were not identified. The community was also potentially carrying out heterotrophic sulfate reduction by a Desulfotalea-like population (52) and either heterotrophic or hydrogen-fed sulfite reduction by Desantisbacteria (51). Many populations were likely involved in recycling nutrients by proteolysis, including Firmicutes and unknown microorganisms. Finally, a flagellum-dependent cell motility was assigned to Planctomycetes and Actinobacteria. Motility is suggested to be lost in highly oligotrophic environments, which further reinforces that the shallower community had a higher input of organic carbon. Finally, the general higher activity of the cells in the MM water was also reflected by mRNA transcripts for transcription.

### Conclusions.

Previous metabolic reconstructions based on MAGs from deep continental groundwaters suggest that the communities host very different “metabolic biomes” dependent on the availability of carbon and energy (24). As shown in this metatranscriptomic study, the more modern and shallower water supported a mixed community with the genetic potential for an array of metabolic strategies. The active cellular processes included DNA replication, transcription, regulation of cell growth, and cell motility. In contrast, the extremely oligotrophic old saline water appeared to preclude the production of mRNA transcripts related to DNA replication and regulation of cell growth. Therefore, although the ultraoligotrophic “old saline” water supported life, it was likely in extreme “slow motion,” with the community maintaining initiation of transcription/translation as an evolutionary “hedge strategy” to rapidly take advantage of any electron donor inputs that may occur.

Many of the SSU rRNA and mRNA transcripts could not be assigned, and the deep phylogenetic placement of some contigs in Fig. 3 suggests there is still the potential for an active deep biosphere diversity waiting to be discovered. Ongoing work with MAGs and single amplified genomes will hopefully help resolve this lacuna in our knowledge.

## MATERIALS AND METHODS

The experimental procedures and analyses are summarized below, and additional details are available in Text S1 in the supplemental material.

10.1128/mBio.01792-18.1 TEXT S1 Supplemental methods. Download Text S1, DOCX file, 0.1 MB. Copyright © 2018 Lopez-Fernandez et al. 2018 Lopez-Fernandez et al. This content is distributed under the terms of the Creative Commons Attribution 4.0 International license.

### Borehole water description.

Two groundwaters from the Äspö HRL were chosen for this study (Fig. S1A). SA1229A containing a “modern marine” water influenced by Baltic Sea water (located at 171.26 mbsl [termed MM]) and KA3385A with “old saline” water (448.65 mbsl [termed OS]). These groundwaters carried iron entirely as Fe^2+^, contained dissolved sulfide (HS^−^), had temporally stable chemistry plus δ^18^O, and neutral pH (24). Nevertheless, they had different chemical compositions (Table 1) and could be characterized with regard to origin and age (32). The MM water has a residence time of less than 20 years due to infiltration of organic carbon-rich Baltic Sea water (median dissolved organic carbon concentration of 6.9 mg liter^−1^), while the OS water has a residence time in the range of thousands of years and a median dissolved organic carbon concentration of 1.4 mg liter^−1^ (24).

**TABLE 1:** Chemical composition of the two sampled groundwaters at different dates during the sampling

Parameter^*a*^	Result for groundwater from sampling date shown^*b*^					
	SA1229A-1			KA3385A-1R		
	18/05/2015	16/11/2015	09/05/2016	26/05/2015	11/11/2015	09/05/2016
Na (mg/liter)	1,510.0	1,530.0	1,740.0	2,450.0	2,520.0	2,520.0
K (mg/liter)	29.60	28.50	27.40	10.90	10.80	10.10
Ca (mg/liter)	281.0	330.0	309.0	2,130.0	2,540.0	2,290.0
Mg (mg/liter)	139.00	146.00	140.00	61.90	61.90	58.20
Cl (mg/liter)	3,120.0	3,139.0	3,103.0	7,314.0	7,502.0	7,489.0
SO_4_ (mg/liter)	267.00	276.00	267.60	406.20	388.80	407.00
SO_4__S (mg/liter)	92.80	99.00	90.50	138.00	152.00	141.00
Br (mg/liter)	11.700	13.100	12.900	45.300	45.300	48.600
F (mg/liter)	1.37	1.43	1.31	1.42	1.46	1.45
Si (mg/liter)	7.18	7.34	6.81	5.45	5.53	5.09
Fe, total (mg/liter)	1.800	1.810	1.790	0.210	0.190	0.190
Fe^2+^ (mg/liter)	1.780	1.810	1.800	0.200	0.190	0.190
Mn (mg/liter)	0.73600	0.80200	0.75900	0.42700	0.43800	0.39600
Li (mg/liter)	0.1070	0.1050	0.1280	1.6600	1.5300	1.6800
Sr (mg/liter)	4.780	5.000	5.040	39.200	39.700	39.800
I (mg/liter)	0.5720	0.5090	0.5980	0.6770	0.5530	0.6320
pH units	7.32	7.34	7.30	7.50	7.51	7.53
EC (mS/m)	987.0	993.0	1,002.0	2,051.0	2,083.0	2,093.0
Drill water (%)	0.50	0.80	0.90	0.20	0.30	0.30
TOC (mg/liter)	6.5	6.9	6.4	1.3	1.4	1.3
DOC (mg/liter)	6.5	6.9	6.3	1.3	1.2	1.3
S_2__HS (mg/liter)	0.07	0.06	0.07	−0.02	−0.02	−0.02
NO_2__N (mg/liter)	0.0004	−0.0002	0.0004	−0.0002	−0.0002	−0.0002
NH_4__N (mg/liter)	5.3500	5.5200	5.0600	0.0800	0.0700	0.0600
PO_4__P (mg/liter)	0.0055	0.0085	0.0061	−0.0005	−0.0005	−0.0005

a Abbreviations: EC, electrical conductivity; TOC, total organic carbon; DOC, dissolved organic carbon.

b Sampling dates are formatted as day/month/year.

### RNA sampling.

Before collection of each sample, borehole water was flushed for three to five section volumes to discard standing borehole water that can be affected by the materials used to enclose the targeted fissure (i.e., the materials can be electron donors for the microorganisms [53]). Samples were collected using an RNA sampling device with a built-in fixation system based upon the concept used for sampling deep marine waters (54) that was constructed by Maskinteknik AB, Oskarshamn, Sweden (Fig. S1B). After being flushed with borehole water under *in situ* temperature and pressure, the groundwater was isolated from the borehole, and the cells were immediately fixed with 5% (vol/vol) water-saturated phenol in absolute ethanol (54). The pressure was then released, and planktonic cells were collected on sterile polyvinylidene fluoride (PVDF), hydrophilic, 0.1-µm, 47-mm Durapore membrane filters (Merck Millipore). Filters with a 0.1-µm pore size were used due to high representation of cells of small size (i.e., <0.22 µm) in this deep environment (24). After filtration, the filter was aseptically transferred to a sterile cryogenic tube (Thermo Scientific), immediately frozen in liquid nitrogen, and transported to the laboratory. Samples were stored at −80°C until further processing. Two biological replicates were collected from each water type (OS1 and OS2 from the “old saline” water and MM1 and MM2 from the “modern marine” water) from June 2015 to March 2016.

### DNA sampling.

Planktonic cells were collected on sterile 0.1-µm membrane filters under *in situ* conditions as described by Lopez-Fernandez et al. (23) (see Table S4A in the supplemental material). Triplicate samples were collected (OS1 to OS3 and MM1 to MM3) from January to April 2016.

10.1128/mBio.01792-18.6 TABLE S4 Sampling and sequencing information of the two water types related to (A) the DNA extraction and 16S rRNA gene tag sequencing, representing the total volume of water, DNA extracted, number of read pairs obtained from the sequencing facility, after merging and quality trimming, and the amount of operational taxonomic units (OTUs), and (B) the RNA extraction and cDNA synthesis giving the volume of water fixed, RNA extracted, cDNA produced, total reads, and quality data. Download Table S4, DOCX file, 0.1 MB. Copyright © 2018 Lopez-Fernandez et al. 2018 Lopez-Fernandez et al. This content is distributed under the terms of the Creative Commons Attribution 4.0 International license.

### RNA/DNA extraction and cDNA amplification.

RNA and DNA were extracted from all filters using the Mo Bio PowerWater RNA or DNA isolation kit, respectively, and following the manufacturer’s instructions, except for the final elution, which was in 50 µl. DNase (Thermo Fisher Scientific) was used to remove DNA contamination from the extracted RNA samples. The quantity and quality of the extracted RNA and DNA were analyzed with a Qubit 2.0 fluorometer (Life Technologies) and by agarose gel electrophoresis, respectively (Table S4). The extracted RNA was utilized to generate cDNA using the Ovation RNA-Seq (transcriptome sequencing) system V2 (NuGEN) following the manufacturer’s instructions. Afterwards, the generated cDNA was purified using the Qiagen MinElute reaction cleanup kit. The cDNA quantity and quality were again analyzed as described above (Table S4B).

Several controls were included in this study, including RNA extraction from the kit chemicals, RNA extraction and cDNA negative controls from sterile filters, DNA contamination of the RNA extractions, and cDNA kit chemical negative controls (detailed in Text S1).

### Metatranscriptome library construction and sequencing.

cDNA library preparation and sequencing were performed at the Science for Life Laboratory, Sweden (www.scilifelab.se). Library preparation was carried out using the Illumina HiSeq TruSeq Nano DNA library prep kit for NeoPrep. Samples were sequenced on HiSeq2500 with a 2- by 126-bp setup using HiSeq SBS kit v4 chemistry. Metatranscriptomic sequencing resulted in 30 to 50 Gb of sequence, with an average of 350 Gb per sample. Details of the metatranscriptomic sequencing and assembly are available in Table S4B.

### Metatranscriptome data analysis.

Metatranscriptome reads were quality checked with FastQC v0.7.2. Low-quality end trimming and removal of adapter sequences were performed with Trimmomatic v0.36 (55), retaining reads with a minimum length of 100 bp for downstream analyses. Replicate MM2 was discarded from the analysis due to low sequencing quality. *De novo* assemblies of the small subunit (SSU) of 16S/18S rRNA and mRNA transcripts were performed separately for each metatranscriptome data set. For this purpose, SSU rRNA reads were filtered from total read data sets with cmsearch (56). Domain-level covariance models available on Rfam were used as a reference for cmsearch. SSU rRNA transcripts were assembled with a mixed strategy involving EMIRGE v0.61, Transabyss v1.5.1, and Minimus v3.1.0 and clustered at a 97% similarity threshold with CD-HIT-EST.

Phylogenetic identification of SSU rRNA contigs was performed with the RAxML Evolutionary Placement Algorithm (RAxML-EPA [57]) using a reference phylogeny, including SSU sequences from the whole tree of life with newly described phyla and from the candidate phyla radiation. Phylogenetic placements were displayed through the web-based iTOL platform (58) and are shown in Fig. 2 (aggregated at the phylum level) and Data Set S1. Abundances of reconstructed SSU sequences were calculated by mapping SSU reads back on SSU sequences with bowtie2 (59) and are reported at the phylum level (Fig. 2; Table S1). Abundances of reconstructed SSU sequences were also used to calculate three alpha-diversity measures (Shannon, Simpson, and inverted Simpson) with the R package vegan (reported in Table S5 in the supplemental material).

10.1128/mBio.01792-18.7 TABLE S5 Alpha-diversity based on the 16S rRNA gene tag sequencing and SSU rRNA transcripts. Download Table S5, DOCX file, 0.1 MB. Copyright © 2018 Lopez-Fernandez et al. 2018 Lopez-Fernandez et al. This content is distributed under the terms of the Creative Commons Attribution 4.0 International license.

mRNA contigs were assembled with Trinity v2.4.0 and annotated with the standalone version of the Interproscan pipeline, including annotations from Gene Ontology (GO). Taxonomic assignment of mRNA transcripts was performed with Kaiju v1.4.2 using a reference database, including all protein sequences from prokaryotes and microbial eukaryotes available at the NCBI nonredundant database on May 2017. Quantification of expressed open reading frames (ORFs) was performed with bowtie2 v2.2.9 (59) and htseq-count (60). Counts were then aggregated according to the assigned GO annotation.

### 16S rRNA gene sequencing and data analysis.

The 16S rRNA gene tag sequencing was carried out as previously described (23). Sequencing was carried out at the Science for Life Laboratory, Sweden, on the Illumina MiSeq platform (61). The UPARSE pipeline was used to process the sequences and cluster operational taxonomic units (OTUs) (62). Phylogenetic assignment of OTUs was performed with RAxML-EPA following the same procedure as per the SSU contigs assembled from metatranscriptome data.

### Data availability.

The 16S rRNA gene amplicon and metatranscriptome sequencing data are available in the NCBI BioProject under ID no. PRJNA400688.

10.1128/mBio.01792-18.8 TABLE S6 Functional annotations of mRNA transcripts in GFF format. Download Table S6, XLSX file, 1.2 MB. Copyright © 2018 Lopez-Fernandez et al. 2018 Lopez-Fernandez et al. This content is distributed under the terms of the Creative Commons Attribution 4.0 International license.

10.1128/mBio.01792-18.10 DATA SET S2 Reference multiple alignment containing reconstructed SSU rRNA gene contigs and tag sequences used to perform the RAxML-EPA phylogenetic placement reported in Fig. 2. Download Data Set S2, TXT file, 11.1 MB. Copyright © 2018 Lopez-Fernandez et al. 2018 Lopez-Fernandez et al. This content is distributed under the terms of the Creative Commons Attribution 4.0 International license.
