# Supplementary figures and images for: Metatranscriptomes Reveal That All Three Domains of Life Are Active but Are Dominated by Bacteria in the Fennoscandian Crystalline Granitic Continental Deep Biosphere
Source: mBio. 2018 Nov 20;9(6):e01792-18. doi: 10.1128/mBio.01792-18 (PMC6247080; doi:10.1128/mBio.01792-18)

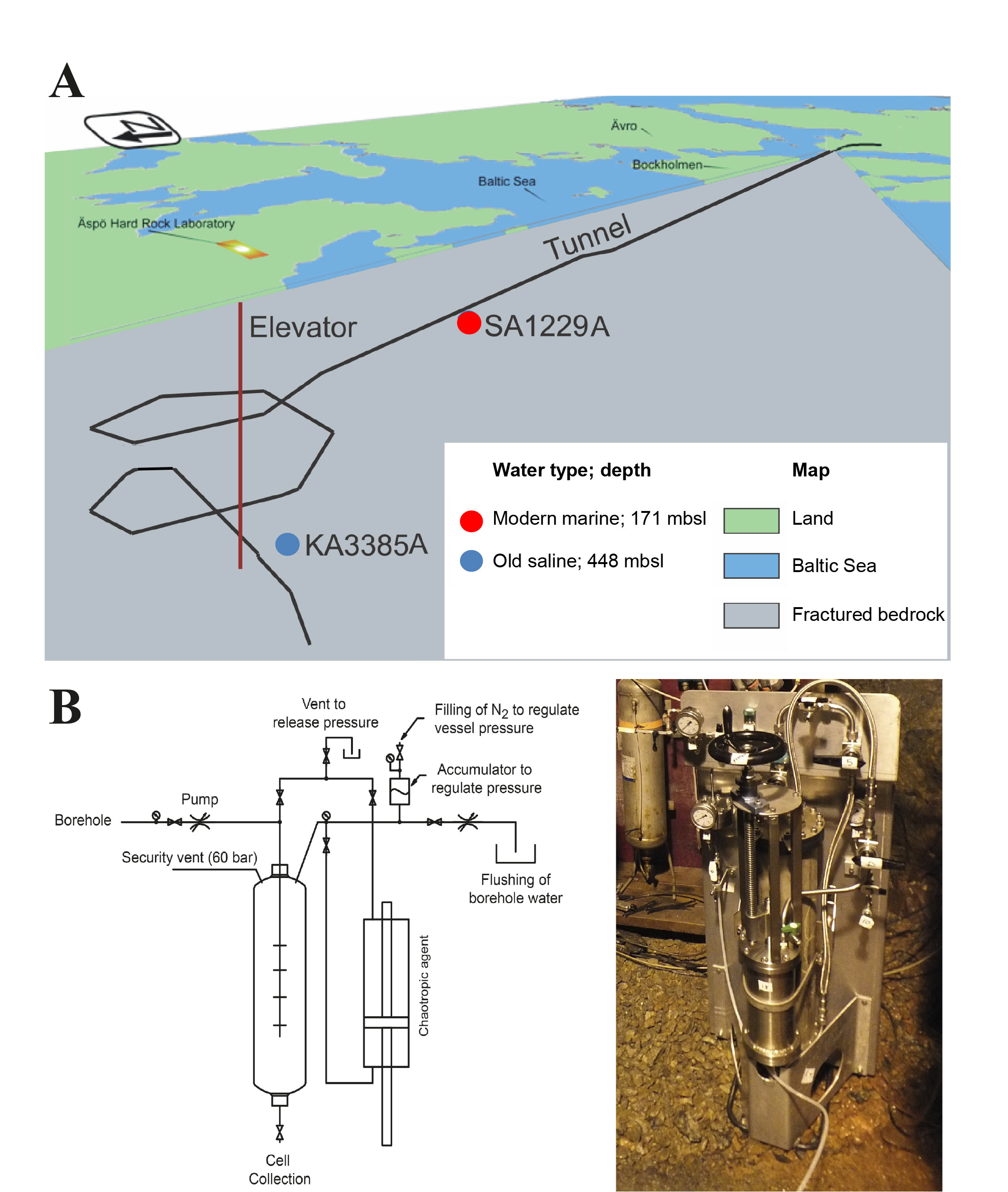

Supplement: FIG S1 [file mbo006184180sf1.tif]
