# Supplementary material for: Metatranscriptomes Reveal That All Three Domains of Life Are Active but Are Dominated by Bacteria in the Fennoscandian Crystalline Granitic Continental Deep Biosphere
Source: mBio. 2018 Nov 20;9(6):e01792-18. doi: 10.1128/mBio.01792-18 (PMC6247080; doi:10.1128/mBio.01792-18)
Supplement: TEXT S1 [file mbo006184180s1.docx]

**Supplementary Methods**

**Borehole water description**

Two groundwaters from the Äspö HRL were chosen for this study (Fig. S1A). SA1229A had high magnesium and potassium concentrations, which are tracers of marine waters in these settings (1) and also had similar values for the conservative variables chloride and δ^18^O as modern Baltic Sea water (2). This groundwater was thus composed of infiltrated brackish marine (Baltic Sea) water and was termed ‘modern marine’ (MM) (3). The precise infiltration age of the groundwater was not known, but was estimated to be < 20 years and is probably even more recent (4). KA3385A had high chloride and calcium concentrations and relatively low δ^18^O values. These features are typical for saline groundwater with a residence time in the order of thousands of years or more (5). However, the chloride concentration of this groundwater was considerably lower than for pure old saline water at the site (2). Therefore, this groundwater had been diluted by waters with lower salinity including glacial meltwater from the retreat of the last Pleistocene continental ice sheet and marine water that intruded during the Littorina Sea stage a few thousands of years ago (2). This groundwater was termed ‘old saline’ (OS). The detailed chemical information during the sampling time is provided in Table 1.

**RNA sampling**

Samples were collected using a RNA sampling device with an in-built fixation system (Fig. S1B). All materials used in the construction were biologically inert. The device has two tanks: one for the stop solution (1 L) and a second tank for the water sample (10 L). After flushing with borehole water under *in situ* temperature and pressure, the groundwater was isolated from the borehole by closing the valve upstream of the sampling device. The cells were then immediately fixed with a stop solution consisting of 5 % (vol/vol) water-saturated phenol in absolute ethanol (6) by using a piston to transfer the stop solution into the main tank. The time taken to fix the isolated planktonic cells in the main tank was less than 10 seconds. The pressure was then released and planktonic cells were collected on sterile polyvinylidene fluoride (PVDF), hydrophilic, 0.1 µm, 47 mm Durapore Membrane Filters (Merck Millipore). When filtration was finished, the filter was aseptically transferred to a sterile cryogenic tube (Thermo Scientific) and immediately frozen in liquid nitrogen. The tubes were transported to the laboratory in liquid nitrogen and stored at -80 °C until further processing. The sampling device was cleaned with absolute ethanol after sampling to avoid possible contamination by completely filling the main tank with approximately 12 L of absolute ethanol, shaking and emptying it three times before collecting a new sample. In addition, all pipes, tubes, tanks and valves were cleaned and/or replaced periodically at Maskinteknik AB, Oskarshamn. No water remained inside the sampling device after filtering the fixed samples and no biofilm growth was detected within the sampling device.

**DNA sampling**

Borehole water was flushed for three to five section volumes to remove stagnant water and ensure pristine groundwater was sampled. Planktonic cells were collected on sterile polyvinylidene fluoride (PVDF), hydrophilic, 0.1 µm, 47 mm Durapore Membrane Filters (Merck Millipore) under *in situ* conditions by connecting a High-Pressure Stainless Steel Filter Holder (Millipore) with a downstream needle valve and pressure gauge directly to the borehole. After filtration of an appropriate volume of water (Table S4A), filters were immediately frozen in liquid nitrogen and transported to the laboratory. Filters were stored at -80 °C until further processing.

**RNA extraction**

Due to the high Fe^2+^ concentration in the MM water type, filters with captured cells from this water were treated before RNA extraction (3). This treatment consisted of adding 1 mL of iron dissolution solution (0.35 M acetic acid, 0.2 M sodium citrate and 0.025 M sodium dithionate) to the filter in a Petri dish, allowing the solution to sit for a few minutes, removing the liquid and washing the filter with phosphate-buffered saline (Amresco). The filter was then placed into a tube, centrifuged for 1 min at 3000 × g, and RNA extracted from the filter using the MO BIO PowerWater RNA as described in the main text.

**Metatranscriptome library construction and sequencing**

cDNA library preparation and sequencing were performed at the Science for Life Laboratory, Sweden (www.scilifelab.se). Library preparation was carried out using the Illumina HiSeq TruSeq Nano DNA Library Prep Kit for NeoPrep. Clustering was done by 'cBot' and samples were sequenced on HiSeq2500 (HiSeq Control Software 2.2.58/RTA 1.18.64) with a 2 × 126 bp setup using 'HiSeq SBS Kit v4' chemistry. The Bcl to FastQ conversion was performed using bcl2fastq-1.8.4 from the CASAVA software suite. The quality scale used was Sanger / phred33 / Illumina 1.8+ (Table S4B).

**Controls of RNA extraction and metatranscriptome sequencing**

Several controls were included in the study that are listed below. 1) RNA and DNA were extracted from the MO BIO PowerWater reagents to test possible contamination from the extraction chemicals. 2) A sterile filter was rolled, placed in a tube, and flash frozen in the Äspö HRL as performed for the fixed samples followed by RNA and DNA extraction and Illumina sequencing library preparation. 3) DNA contamination of the RNA extractions (after DNase treatment) was checked with 40 cycles of PCR amplification using universal 16S rRNA gene primers 27F and 1492R (7). 4) cDNA negative controls were performed using the Ovation^®^ RNA-Seq System V2 with the RNA extracted from the blank filters described in control (2) and the MO BIO PowerWater RNA extraction reagents. 5) Finally, cDNA generation was carried out using only the Ovation^®^ RNA-Seq System V2 reagents to test for contamination from the kit reagents. All controls were negative as nucleic acid concentrations were below the Qubit 2.0 Fluorometer’s detection limit, Illumina sequencing library preparation failed, or no bands from cDNA amplification products were obtained after electrophoresis. All of these controls support that the metatranscriptome sequencing was a true representation of the RNA transcripts within the community.

**Metatranscriptome data analysis**

Metatranscriptome reads were quality-checked with FastQC v0.7.2 (https://www.bioinformatics.babraham.ac.uk). Low quality end trimming and removal of adapter sequences were performed with Trimmomatic v0.36 (8), retaining reads with a minimum length of 100 bp for downstream analyses. Replicate MM2 was discarded from the analysis due to a bad sequencing quality. *De novo* assemblies of SSU (16S/18S) rRNA and mRNA transcripts were performed separately for each metatranscriptome dataset. To this purpose, SSU rRNA reads were filtered from total read datasets with the cmsearch module of the Infernal package v1.1.2 (9), using default parameters. SSU rRNA transcripts were assembled with EMIRGE v0.61 (10) and Transabyss v1.5.1 (11). Contigs obtained from the two assemblers were merged with Minimus v3.1.0 (12). Redundant contigs were removed with CD-HIT-EST v4.6.4 (13) with a similarity threshold of 97 %. The assembly strategy was quality-checked by mapping SSU reads on reconstructed contigs (≥ 300 bp and ≥ 5 average coverage) with bowtie2 v2.2.9 (14). For OS1, OS2, MM1 samples, 78, 88 and 91 % respectively of reads mapped concordantly, according to bowtie2 reports.

Although raw reads have being recently used to perform faster phylogenetic placement (15), in this study the phylogenetic identification was performed with the SSU rRNA contigs by using the Evolutionary Placement Algorithm included in RAxML v8.2.10 (16). Briefly, given a reference multiple alignment (RMA) and the related reference phylogenetic tree (RPT), contigs were aligned to the RMA before being inserted into the RPT by re-optimization of RPT edge lengths through the Maximum Likelihood model implemented in RAxML (Data S2). A RPT including SSU sequences from Hug et al (17) and Anantharaman et al (18) was adopted to perform the phylogenetic identification. The choice of this dataset ensured the use of an up-to-date tree of life including newly described phyla. Only phylogenetic placements supported by a likelihood weight ratio of ≥ 0.90 were retained. Phylogenetic placements were displayed through the web-based iTOL platform (19) and are shown in Fig. 2 & Data S1. Abundances of reconstructed SSU sequences were calculated by mapping SSU reads back on SSU sequences with bowtie2 v2.2.9 (14) and are reported, aggregated at the phylum level, in Fig. 2 and Table S1. Abundances of reconstructed SSU sequences were also used to calculate three alpha-diversity measures (Shannon, Simpson, and Inverted Simpson) with the R package vegan (https://CRAN.R-project.org/package=vegan) and are reported in Table S5.

Assembly of mRNA metatranscriptome reads was performed with Trinity v2.4.0 (20) using default parameters. Taxonomic assignment of assembled transcripts was performed with Kaiju v1.4.2 (21). This classification was also used to select the most appropriate genetic code to translate and annotate the transcripts. Upon taxonomic assignment, the assembled transcripts were translated in all six frames with an in-house script (available at https://github.com/NBISweden/GAAS). For each assembled transcript, the longest open reading frame was retained for downstream analyses. Each set of translated protein sequences was then annotated with the standalone version of the Interproscan pipeline (v5.15) (22), including annotations from Gene Ontology (23) and several pathway databases (KEGG (24) , MetaCyc (25), Reactome (26) and UniPathway (27)) (Table S6).

In order to quantify the expression of annotated ORFs, reads were mapped to assembled transcripts with bowtie2 v2.2.9 (14) and counted with htseq-count v0.9.1 (28). For each transcript, counts were then aggregated according to the assigned GO annotation.

**16S rRNA gene sequencing and data analysis**

The 16S rRNA gene tag sequencing was carried as previously described (29). Briefly, region V3-V4 of the 16S rRNA gene was amplified utilizing primers 341F and 805R (30) according to published procedures (31). Sequencing was carried out at the Science for Life Laboratory, Sweden (www.scilifelab.se) on the Illumina MiSeq platform as previously described (32, 33). The UPARSE pipeline was used to process the sequences and cluster operational taxonomic units (OTUs) (34). Phylogenetic assignment of OTUs was performed with RAxML-EPA following the same procedure as per the SSU contigs assembled from metatranscriptome data.

**References**

1. Gimeno MJ, Auqué LF, Acero P, Gómez JB. 2014. Hydrogeochemical characterisation and modelling of groundwaters in a potential geological repository for spent nuclear fuel in crystalline rocks (Laxemar, Sweden). Applied Geochemistry 45:50-71.

2. Mathurin FA, Åström ME, Laaksoharju M, Kalinowski BE, Tullborg EL. 2012. Effect of tunnel excavation on source and mixing of groundwater in a coastal granitoidic fracture network. Environmental Science and Technology 46:12779-12786.

3. Wu X, Holmfeldt K, Hubalek V, Lundin D, Åström M, Bertilsson S, Dopson M. 2015. Microbial metagenomes from three aquifers in the Fennoscandian shield terrestrial deep biosphere reveal metabolic partitioning among populations. The ISME Journal 10(5):1192-1203.

4. Mathurin FA, Drake H, Tullborg EL, Berger T, Peltola P, Kalinowski BE, Åström ME. 2014. High cesium concentrations in groundwater in the upper 1.2km of fractured crystalline rock - Influence of groundwater origin and secondary minerals. Geochimica et Cosmochimica Acta 132:187-213.

5. Louvat D, Michelot JL, Aranyossy JF. 1999. Origin and residence time of salinity in the Aspo groundwater system. Applied Geochemistry 14:917-925.

6. Feike J, Jürgens K, Hollibaugh JT, Krüger S, Jost G, Labrenz M. 2012. Measuring unbiased metatranscriptomics in suboxic waters of the central Baltic Sea using a new in situ fixation system. The ISME Journal 6:461-470.

7. Stackebrandt E, Goodfellow M. 1991. Nucleic acid techniques in bacterial systematics. Wiley.

8. Bolger AM, Lohse M, Usadel B. 2014. Trimmomatic: a flexible trimmer for Illumina sequence data. Bioinformatics 30:2114-2120.

9. Nawrocki EP, Eddy SR. 2013. Infernal 1.1: 100-fold faster RNA homology searches. Bioinformatics 29:2933-2935.

10. Miller CS, Baker BJ, Thomas BC, Singer SW, Banfield JF. 2011. EMIRGE: reconstruction of full-length ribosomal genes from microbial community short read sequencing data. Genome Biology 12:R44.

11. Robertson G, Schein J, Chiu R, Corbett R, Field M, Jackman SD, Mungall K, Lee S, Okada HM, Qian JQ. 2010. De novo assembly and analysis of RNA-seq data. Nature Methods 7:909-912.

12. Sommer DD, Delcher AL, Salzberg SL, Pop M. 2007. Minimus: a fast, lightweight genome assembler. BMC Bioinformatics 8:64.

13. Fu L, Niu B, Zhu Z, Wu S, Li W. 2012. CD-HIT: accelerated for clustering the next-generation sequencing data. Bioinformatics 28:3150-3152.

14. Langmead B, Trapnell C, Pop M, Salzberg SL. 2009. Ultrafast and memory-efficient alignment of short DNA sequences to the human genome. Genome Biology 10:R25.

15. Barbera P, Kozlov AM, Czech L, Morel B, Darriba D, Flouri T, Stamatakis A. 2018. EPA-ng: massively parallel evolutionary placement of genetic sequences. BioRxiv:291658.

16. Berger SA, Krompass D, Stamatakis A. 2011. Performance, accuracy, and web server for evolutionary placement of short sequence reads under maximum likelihood. Systematic Biology 60:291-302.

17. Hug LA, Baker BJ, Anantharaman K, Brown CT, Probst AJ, Castelle CJ, Butterfield CN, Hernsdorf AW, Amano Y, Ise K. 2016. A new view of the tree of life. Nature Microbiology 1:16048.

18. Anantharaman K, Brown CT, Hug LA, Sharon I, Castelle CJ, Probst AJ, Thomas BC, Singh A, Wilkins MJ, Karaoz U, Brodie EL, Williams KH, Hubbard SS, Banfield JF. 2016. Thousands of microbial genomes shed light on interconnected biogeochemical processes in an aquifer system. Nature Communications 7:13219.

19. Letunic I, Bork P. 2016. Interactive tree of life (iTOL) v3: an online tool for the display and annotation of phylogenetic and other trees. Nucleic Acids Research 44:W242-W245.

20. Grabherr MG, Haas BJ, Yassour M, Levin JZ, Thompson DA, Amit I, Adiconis X, Fan L, Raychowdhury R, Zeng Q. 2011. Full-length transcriptome assembly from RNA-Seq data without a reference genome. Nature Biotechnology 29:644-652.

21. Menzel P, Ng KL, Krogh A. 2016. Fast and sensitive taxonomic classification for metagenomics with Kaiju. Nature Communications 7:11257.

22. Jones P, Binns D, Chang H-Y, Fraser M, Li W, McAnulla C, McWilliam H, Maslen J, Mitchell A, Nuka G. 2014. InterProScan 5: genome-scale protein function classification. Bioinformatics 30:1236-1240.

23. Consortium GO. 2014. Gene ontology consortium: going forward. Nucleic Acids Research 43:D1049-D1056.

24. Ogata H, Goto S, Sato K, Fujibuchi W, Bono H, Kanehisa M. 1999. KEGG: Kyoto encyclopedia of genes and genomes. Nucleic Acids Research 27:29-34.

25. Caspi R, Foerster H, Fulcher CA, Hopkinson R, Ingraham J, Kaipa P, Krummenacker M, Paley S, Pick J, Rhee SY. 2006. MetaCyc: a multiorganism database of metabolic pathways and enzymes. Nucleic Acids Research 34:D511-D516.

26. Stein LD. 2004. Using the Reactome database. Current Protocols in Bioinformatics 38(1):8-7.

27. Morgat A, Coissac E, Coudert E, Axelsen KB, Keller G, Bairoch A, Bridge A, Bougueleret L, Xenarios I, Viari A. 2011. UniPathway: a resource for the exploration and annotation of metabolic pathways. Nucleic Acids Research 40:D761-D769.

28. Anders S, Pyl PT, Huber W. 2015. HTSeq—a Python framework to work with high-throughput sequencing data. Bioinformatics 31:166-169.

29. Lopez-Fernandez M, Broman E, Turner S, Wu X, Bertilsson S, Dopson M. 2018. Investigation of viable taxa in the deep terrestrial biosphere suggests high rates of nutrient recycling. FEMS Microbiology Ecology 94(8):fiy121.

30. Herlemann DP, Labrenz M, Jurgens K, Bertilsson S, Waniek JJ, Andersson AF. 2011. Transitions in bacterial communities along the 2000 km salinity gradient of the Baltic Sea. The ISME Journal 5:1571-1579.

31. Wickham H. 2009. Positioning, p 115-137, ggplot2. Springer.

32. Hugerth LW, Wefer HA, Lundin S, Jakobsson HE, Lindberg M, Rodin S, Engstrand L, Andersson AF. 2014. DegePrime, a program for degenerate primer design for broad-taxonomic-range PCR in microbial ecology studies. Applied Environmental Microbiology 80:5116-5123.

33. Lindh MV, Figueroa D, Sjostedt J, Baltar F, Lundin D, Andersson A, Legrand C, Pinhassi J. 2015. Transplant experiments uncover Baltic Sea basin-specific responses in bacterioplankton community composition and metabolic activities. Frontiers in Microbiology 6:223.

34. Edgar RC. 2013. UPARSE: highly accurate OTU sequences from microbial amplicon reads. Nature Methods 10:996-998.
