# Supplementary material for: Metatranscriptomes Reveal That All Three Domains of Life Are Active but Are Dominated by Bacteria in the Fennoscandian Crystalline Granitic Continental Deep Biosphere
Source: mBio. 2018 Nov 20;9(6):e01792-18. doi: 10.1128/mBio.01792-18 (PMC6247080; doi:10.1128/mBio.01792-18)
Supplement: TABLE S4 [file mbo006184180st4.docx]

**Table S4.** Sampling and sequencing information of the two water types related to A) the DNA extraction and 16S rRNA gene tag sequencing, representing the total volume of water, DNA extracted, number of read pairs obtained from the sequencing facility, after merging and quality trimming, and the amount of Operational Taxonomic Units (OTUs); and B) the RNA extraction and cDNA synthesis giving the volume of water fixed, RNA extracted, cDNA produced, total reads and quality data.

**A**

| Sample ID | Sampling site | Water type | Total water volume (L) | DNA (ng/µL) | | Read pairs (2×301 bp) | | Reads after merging | | Reads after quality trimming | | Amount of OTUs | | |
| --- | --- | --- | --- | --- | --- | --- | --- | --- | --- | --- | --- | --- | --- | --- |
| **DNA sampling** | |  |  |  | |  | |  | |  | |  | | |
| MM1 | SA1229A | MM | 11 | 1.2 | | 213133 | | 134161 | | 77449 | | 997 | | |
| MM2 | SA1229A | MM | 29 | 3.0 | | 296575 | | 188489 | | 106916 | | 746 | | |
| MM3 | SA1229A | MM | 14 | 2.4 | | 185548 | | 116980 | | 66265 | | 622 | | |
| OS1 | KA3385A | OS | 23 | 0.7 | | 81483 | | 52308 | | 29850 | | 764 | | |
| OS2 | KA3385A | OS | 28 | 0.3 | | 464299 | | 300458 | | 167579 | | 1081 | | |
| OS3 | KA3385A | OS | 35 | 0.3 | | 129391 | | 81871 | | 47361 | | 863 | | |
| **Negative controls** | | | | | | | | | | |  | |  |  |
| Control-1^a^ | SA1229A | - | - | | BDL^d^ | | - | | - | | - | | - |  |
| Control-2^b^ | KA3385A | - | - | | BDL | | - | | - | | - | | - |  |
| Control-3^c^ | - | - | - | | BDL | | - | | - | | - | | - |  |

^a^Control-1: DNA extracted from a blank filter collected under the same conditions as the samples at borehole site SA1229A. DNA was checked by Polymerase Chain Reaction (PCR), but no product was obtained.

^b^Control-2: DNA extracted from a blank filter collected under the same conditions as the samples at borehole site KA3385A. DNA was checked by Polymerase Chain Reaction (PCR), but no product was obtained.

^c^Control-3: DNA extracted from the extraction kit reagents.

^d^Below detection limit

**B**

| Sample ID | Sampling site | | Water type | Filters |  | Total water volume (L) | | RNA (ng/µL) | cDNA (ng/µL) | Mreads^a^ | >=Q30^b^ | Avg. FS^c^ | |
| --- | --- | --- | --- | --- | --- | --- | --- | --- | --- | --- | --- | --- | --- |
|  | | **RNA Sampling device** | | | | | | | |  |  |  | |
| MM1 | SA1229A | | MM^d^ | 18 |  | 180 | | 0.02 | 60 | 50.04 | 96.31 | 350 | |
| MM2^e^ | SA1229A | | MM | 11 |  | 110 | | 0.02 | 60 | - | - | - | |
| OS1 | KA3385A | | OS^f^ | 14 |  | 140 | | 0.02 | 39 | 30.04 | 95.8 | 350 | |
| OS2 | KA3385A | | OS | 15 |  | 150 | | 0.02 | 33 | 34.96 | 91.12 | 350 | |
|  | | **Negative controls** | | | | | | | |  |  |  | |
| Control-1^g^ | SA1229A | | - | 1 |  | - | | BDL^j^ | BDL | - | - | - | |
| Control-2^h^ | KA3385A | | - | 1 |  | - | | BDL | BDL | - | - | - | |
| Control-3^i^ | - | | - | - |  | | - | BDL | BDL | - | - | | - |

^a^Mreads: Total million reads for a sample

^b^>=Q30: Aggregated percentage of bases that have quality score more than Q30

^c^Avg. FS: Average fragment size of the library

^d^MM: Modern marine

^e^MM2: Discarded biological replicate of the modern marine water

^f^OS: Old saline

^g^ Control-1: RNA extracted from a blank filter collected under the same conditions as the samples at borehole site SA1229A. Total RNA was sent for sequencing, but no library was obtained.

^h^Control-2: RNA extracted from a blank filter collected under the same conditions as the samples at borehole site KA3385A. Total RNA was sent for sequencing, but no library was obtained.

^i^Control-3: RNA extracted from the extraction kit reagents.

^j^Below detection limit
