# Supplementary material for: Metatranscriptomes Reveal That All Three Domains of Life Are Active but Are Dominated by Bacteria in the Fennoscandian Crystalline Granitic Continental Deep Biosphere
Source: mBio. 2018 Nov 20;9(6):e01792-18. doi: 10.1128/mBio.01792-18 (PMC6247080; doi:10.1128/mBio.01792-18)
Supplement: TABLE S5 [file mbo006184180st5.docx]

**Table S5.** Alpha diversity based on the 16S rRNA gene tag sequencing and SSU rRNA transcripts.

|  | Shannon | Simpson | Inversed Simpson |
| --- | --- | --- | --- |
| **16S rRNA gene tag sequencing** |  |  |  |
| OS1 | 3.52 | 0.93 | 14.36 |
| OS2 | 3.42 | 0.93 | 14.91 |
| OS3 | 3.53 | 0.94 | 18.22 |
| MM1 | 3.92 | 0.94 | 16.97 |
| MM2 | 3.15 | 0.89 | 9.82 |
| MM3 | 2.96 | 0.89 | 9.46 |
|  |  |  |  |
| **16S rRNA transcripts** |  |  |  |
| OS1 | 6.05 | 0.99 | 185.0 |
| OS2 | 5.56 | 0.99 | 113.8 |
| MM1 | 6.66 | 0.99 | 190.1 |
